# β-catenin in epithelial tumorigenesis

**DOI:** 10.18632/aging.100783

**Published:** 2015-07-17

**Authors:** Tsai-Ling Lu, Chun-Ming Chen

**Affiliations:** Department of Life Sciences and Institute of Genome Sciences, National Yang-Ming University, Taipei, Taiwan

β-catenin is a critical mediator of the Wnt canonical pathway for multiple cellular responses. Using transgenic mice, loss or activation of β-catenin has been studied in multiple tissues to elucidate its role *in vivo*. In this editorial, we focus on recent findings examining the involvement of β-catenin in cancer initiation and progression of epithelial tissues in thymus and prostate.

**Figure 1:**
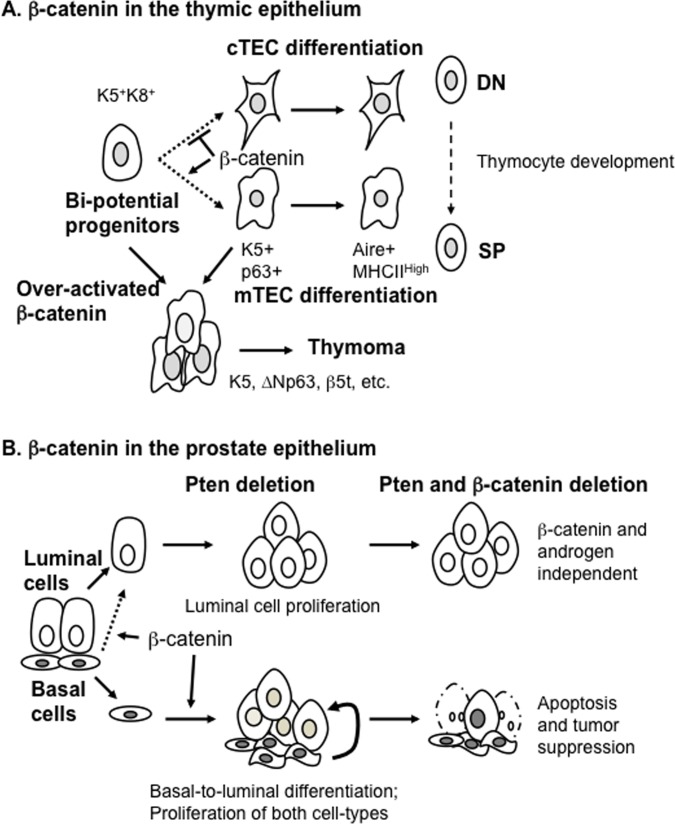
The proposed models for the functions of β-catenin in epithelial differentiation and tumorigenesis of thymus and prostate (**A**) β-catenin is critical for maintaining mTEC differentiation and preventing cTEC differentiation of bi-potent progenitors, whereas over-activated β-catenin drives thymoma initiation and progression. (**B**) β-catenin is required for basal-to-luminal differentiation and basal-derived prostate cancer progression. In contrast, β-catenin is dispensable for luminal-derived tumors.

*β-catenin in the thymic epithelium and thymoma*. In thymus, correct differentiation of cortical thymic epithelial cells (cTECs) and medullary TECs (mTECs) is required for thymocyte development and selection. Ablation of β-catenin in Keratin 5 (K5)-expressing mTECs results in thymic atrophy, mainly due to a defective IL-7 niche in β-catenin-deficient mTEC derivatives that are unable to support early thymocyte development [1]. Using a lineage-tracing approach, we found that an increased number of K8-expressing cTECs are generated from β-catenin-deficient mTECs, and differentiation of Aire^+^ and MHCII^high^ mTECs from β-catenin-deficient mTECs is defective [1]. Additionally, we created transgenic mouse lines, which expressed an active form of β-catenin fused with the ligand-binding domain of the estrogen receptor (∆N64Ctnnb1/ER^T2^) in mTECs, that could functionally rescue defects in thymopoiesis caused by β-catenin-deficient mTEC derivatives [2]. These findings suggest that endogenous β-catenin is required for maintaining mTEC differentiation and may block cTEC differentiation (Figure 1A).

In addition, expression of the ∆N64Ctnnb1/ER^T2^ transgene in mTECs can activate Wnt/β-catenin canonical signaling and drive thymoma initiation and progression [2]. Early thymoma lesions driven by the ∆N64Ctnnb1/ER^T2^ transgene are frequently identified at the cortical-medullary junction (CMJ) of the transgenic thymi. Interestingly, the CMJ of the thymus is hypothesized to be the anatomic location of bi-potential progenitors that can give rise to both cTECs and mTECs. Thus, this observation implies that the thymic epithelial progenitors at CMJ are susceptible to β-catenin oncogenic signaling during thymoma development. The thymoma lesions express K5, ∆Np63 (α and β isoforms), and β5t, which is a protease subunit used as a differential diagnostic marker of human type B3 thymomas. Consistent with expression patterns found in human thymomas, ∆N64Ctnnb1/ER^T2-^mediated thymomas showed loss of AIRE and downregulation of p21. Moreover, histologic characteristics of ∆N64Ctnnb1/ER^T2^ transgene-induced thymomas have a squamoid appearance and loose connective tissue in the perivascular space resembling that of human type B3 thymomas (or atypical thymomas). Thus, our recent findings demonstrate that β-catenin oncogenic signaling drives K5-expressing thymoma initiation and progression (Figure 1A).

*β-catenin in the prostate epithelial lineages and cancer*. Basal and luminal epithelial cells are two major cell types of the prostate gland. Both luminal and basal cells in the adult prostate are largely generated from their corresponding cell lineages [3-5]. At the adult stage, approximately 2% of luminal cells can be generated from prostate basal cells under intact and prostate regeneration conditions using lineage-tracing approaches [3, 4]. β-catenin ablation in prostate luminal cells is not required for prostate epithelial homeostasis [6]. Interestingly, specific ablation of β-catenin in prostate basal cells blocks basal-to-luminal differentiation, indicating a critical role of β-catenin in this process [7] (Figure 1B).

*PTEN* is another gene commonly mutated or lost in prostate cancer. Conditional ablation of *Pten* in prostate luminal cells in mice confirms that Pten acts as a tumor suppressor in prostate cancer progression. Upon conditional ablation of the *Pten* gene in prostate basal cells, basal-to-luminal differentiation is promoted, which generates neoplastic luminal cells [3, 4]. These findings suggest both prostate luminal cells and basal cells can act as cells of origin for prostate cancer initiation. However, findings regarding aggressiveness of tumor cells generated from luminal cells versus basal cells remain controversial [3-5]. To further investigate the invasiveness of prostate cancer originating from basal cells versus luminal cells, we characterized the epithelial-mesenchymal transition (EMT)-inducing transcription factors (TFs) and tumor-initiating Lin^−^Sca-1^+^CD49f^+^ (LSC) stem/progenitors in basal- and luminal-derived Pten-deficient prostate cancer, respectively. Our results show that aggressive cancer-associated EMT-TFs and LSCs are higher in basal-derived prostate cancer cells compared with luminal-derived cancer cells [7]. We further demonstrate that loss of β-catenin suppresses basal-derived prostate cancer progression but is dispensable for luminal-derived prostate cancer [7]. Therefore, our recent findings suggest that β-catenin is differentially required for Pten-deficient prostate cancer arising from basal cells compared with that from luminal cells (Figure 1B).

Taken together, our findings indicate that Wnt/β-catenin canonical signaling is essential for cellular differentiation of bi-potent epithelial progenitors in thymus and prostate. Aberrant β-catenin activation in the K5-expressing epithelial derivatives of the thymus and prostate gland promotes tumor progression.
